# Transforming growth factor β1 enhances heme oxygenase 1 expression in human synovial fibroblasts by inhibiting microRNA 519b synthesis

**DOI:** 10.1371/journal.pone.0176052

**Published:** 2017-04-19

**Authors:** Shu-Jui Kuo, Wei-Hung Yang, Shan-Chi Liu, Chun-Hao Tsai, Horng-Chaung Hsu, Chih-Hsin Tang

**Affiliations:** 1 Graduate Institute of Clinical Medical Science, China Medical University, Taichung, Taiwan; 2 Department of Orthopedic Surgery, China Medical University Hospital, Taichung, Taiwan; 3 Department of Orthopedic Surgery, Taichung Hospital, Ministry of Health and Welfare, Taichung, Taiwan; 4 School of Chinese Medicine, China Medical University, Taichung, Taiwan; 5 Department of Nursing, National Taichung University of Science and Technology, Taichung, Taiwan; 6 Graduate Institute of Biotechnology, National Chung Hsing University, Taichung, Taiwan; 7 Graduate Institute of Basic Medical Science, China Medical University, Taichung, Taiwan; 8 Department of Pharmacology, School of Medicine, China Medical University, Taichung, Taiwan; 9 Department of Biotechnology, College of Health Science, Asia University, Taichung, Taiwan; Chang Gung University, TAIWAN

## Abstract

**Background:**

Osteoarthritis (OA) is manifested by synovial inflammation and cartilage destruction that is directly linked to synovitis, joint swelling and pain. In the light of the role of synovium in the pathogenesis and the symptoms of OA, synovium-targeted therapy is a promising strategy to mitigate the symptoms and progression of OA. Transforming growth factor beta 1 (TGF-β1), a secreted homodimeric protein, possesses unique and potent anti-inflammatory and immune-regulatory properties in many cell types. Heme oxygenase 1 (HO-1) is an inducible anti-inflammatory and stress responsive enzyme that has been proven to prevent injuries caused by many diseases. Despite the similar anti-inflammatory profile and their involvement in the pathogenesis of arthritic diseases, no studies have as yet explored the possibility of any association between the expression of TGF-β1 and HO-1.

**Methodology/Principal findings:**

TGF-β1-induced HO-1 expression was examined by HO-1 promoter assay, qPCR, and Western blotting. The siRNAs and enzyme inhibitors were utilized to determine the intermediate involved in the signal transduction pathway. We showed that TGF-β1 stimulated the synthesis of HO-1 in a concentration- and time-dependent manner, which can be mitigated by blockade of the phospholipase (PLC)γ/protein kinase C alpha (PKC)α pathway. We also showed that the expression of miRNA-519b, which blocks HO-1 transcription, is inhibited by TGF-β1, and the suppression of miRNA 519b could be reversed via blockade of the PLCγ/PKCα pathway.

**Conclusions/Significance:**

TGF-β1 stimulated the expression of HO-1 via activating the PLCγ/PKCα pathway and suppressing the downstream expression of miRNA-519b. These results may shed light on the pathogenesis and treatment of OA.

## Introduction

Osteoarthritis (OA) is manifested by synovial inflammation and cartilage destruction that correlates with synovitis, joint swelling and pain. The inflammation of the synovium can be seen throughout the whole course of OA. The pro-inflammatory and catabolic mediators synthesized by the inflamed synovium led to the excessive synthesis of the proteolytic enzymes that are responsible for cartilage destruction. Cartilage breakdown will amplify synovial inflammation, thus creating a vicious cycle. Previous studies have demonstrated that OA synovial cells are vital in maintaining arthritic pathologies by producing matrix degradation enzymes and inflammatory mediators [1, 2]. In the light of the role of synovium in the pathogenesis and the symptoms of OA, synovium-targeted therapy may potentially halt the progression of structural destruction and mitigate the symptoms of the disease [3].

Transforming growth factor beta 1 (TGF-β1) is a secreted homodimeric protein that exerts its effect by binding to TGF-β type I (TGFBR1) and type II (TGFBR2) serine/threonine kinase receptors on the surface of cell membrane[4]. TGF-β1 has been recognized to have unique and potent anti-inflammatory and immune-regulatory properties in many cell types[5, 6]. In OA, TGF-β1 induces synovial fibrosis and also cartilage anabolism [7]. However, there is no clear evidence as to the role that TGF-β1 may play in alleviating the inflammatory response in OA pathogenesis.

Heme oxygenase 1 (HO-1) is an inducible anti-inflammatory and stress responsive enzyme that can convert the cytotoxic heme into the catabolites without cytotoxicity, including biliverdin, carbon monoxide and iron[8]. The synthesis of HO-1 can be induced by not only cytokines and growth factors but also heme (its substrate) and antioxidants. HO-1 and heme catabolism have been proven to prevent injuries caused by many diseases [9–11]. Moreover, induction of HO-1 expression alleviates arthritic severity in animal models [12].

Micro-ribonucleic acids (miRNAs) are non-coding single-stranded RNAs containing about 20 nucleotides that modulate the expression of target genes in the post-transcriptional level[13]. They modulate the expression of target gene in the post-transcriptional level via base-pairing with the seed sequence of target mRNA molecules, the so-called 3’-untranslated region (3’-UTR), thus inhibiting the expression of target genes. A multitude of miRNAs are recognized as being involved in OA pathogenesis [14, 15].

Despite their similar anti-inflammatory profile and involvement in the pathogenesis of arthritic diseases, no research has as yet investigated the possible correlation between TGF-β1 and HO-1 activity. We therefore sought to determine the anti-inflammatory potential of TGF-β1 in human synovial cells, the way in which TGF-β1 and HO-1 affect each other, and to define how miRNA mediates the activity of the TGF-β1-HO-1 pathway. We hypothesized that TGF-β1 may upregulate HO-1 expression via modulation of intermediate miRNA expression.

## Materials and methods

### Materials

The reagent Trizol, Lipofectamine 2000 transfection reagent, miR-519b mimic, and control miRNA were purchased from Life Technologies (Carlsbad, CA). Recombinant human TGF-β1 and the TGF-β1 ELISA kit were bought from PeproTech (Rocky Hill, NJ, USA). The antibodies against phospho PLCγ and phospho PKCα were the products from the Cell Signaling (Danvers, MA, USA). U73122, Gö6976, and GF109203X were bought from Enzo Biochem, Inc. (Enzo, New York, NY). Anti-PLCγ, anti-PKCα, anti-HO-1, anti-mouse and anti-rabbit conjugated horseradish peroxidase (HRP) antibodies were the products from Santa Cruz (Santa Cruz, CA, USA). The small interfering RNAs (siRNAs) against PLCγ, PKCα as well as the control siRNAs were the products from the Dharmacon Research (Lafayette, CO, USA). The pertinent chemicals not mentioned above were purchased from the Sigma-Aldrich (St. Louis, MO, USA).

### Cell culture

Human osteoarthritic synovial fibroblasts (OASFs) were yielded via processing the synovial tissues from the 15 patients undergoing knee replacement surgeries due to end stage knee OA by collagenase. The cells from passages 3 to 6 were utilized for all the pertinent studies. The Institutional Review Board of China Medical University Hospital approved this study. All the details of the study were carried out in accordance with the guidelines and regulations by the Institutional Review Board of the China Medical University Hospital. Informed written consent was obtained from all patients.

### Quantitative real-time PCR analysis

All of the quantitative polymerase chain reaction (qPCR) studies were performed for three times by utilizing the StepOnePlus sequence detection system (Applied Biosystems). Total cDNA was added with HO-1 or GAPDH primer and KAPA SYBR^®^ FAST qPCR Kit (Applied Biosystems, Foster City, CA). The sequences of primers were: F-5’-TGCTCGCATGAACACTCTGGAGAT-3’ and R-5’-ATGGCATAAATTCCCACTGCCACG-3’ for HO-1, and F-5’- AAGCCCATCACCATCTTCCAG-3’ and R-5’-AGGGGCCATCCACAGTCTTCT-3’ for GAPDH [16].

As for the miRNA studies, the cDNAs were synthesized from 100 ng RNA by utilizing a Mir-X^™^ miRNA First-Strand Synthesis Kit (Clontech Laboratories, Palo Alto, CA). The relative gene expression was quantified by referencing upon the endogenous control gene (U6) [17]. The sequence of primer was: F-5’- AAAGTGCATCCTTTTAGAGGTT-3’ for miRNA-519b.

### Western blot analysis

The OASFs were lysed in the radioimmunoprecipitation (RIPA) buffer with protein inhibitors. The extracted proteins were processed by the sodium dodecyl sulfate-polyacrylamide gel electrophoresis (SDS-PAGE) and transferred to the Immobilon polyvinyldifluoride (PVDF) membranes. The transferred blots were subsequently blocked with 4% bovine serum albumin (BSA) for 1 hour at room temperature and then probed with rabbit anti-human primary antibodies against HO-1, PLCγ, and PKCα for 1 hour at room temperature. The blots were subsequently incubated with the donkey anti-rabbit peroxidase-conjugated secondary antibody (1:1000) for 1 hour at room temperature after washing out the primary antibodies for three washes. The enhanced chemiluminescence was finally visualized under an Imagequant LAS 4000 (GE Healthcare, Pewaukee, WI, USA).

### Luciferase reporter assay

The human HO-1 promoter plasmid was gift from Dr. Y.C. Liang (Taipei Medical University, Taipei, Taiwan); the pGL2/hHO3.2-Luc reporter plasmid containing a 3292-bp fragment -3106 to +186 relative to the transcription start site of the human HO-1 gene. OASFs were co-transfected with 1 μg HO-1-luciferase plasmid and 0.4 μg β-galactosidase expression vector. The collected OASFs were dispersed over the 12-well plates and lysed with reporter lysis buffer 24 hours after transfection. The Dual-luciferase^®^ reporter assay system was utilized to determine the extent of luciferase and renilla activities in the cellular extracts, and the relative luciferase activity was quantified by the ratio of luciferase/renilla activity referenced to the ratio of control samples[16].

### Plasmid construct

The 3′-UTR-luciferase reporter constructs including the 3′-UTR regions of HO-1 with wild-type and mutant binding sites for miRNA-519b were utilized to validate that miRNA-519b inhibits the transcription of HO-1. After amplifying the cDNAs with PCR reaction, the yielded PCR products were cloned into a *pmirGLO* reporter vector (Promega) between the *Nhe*I and *Xho*I restriction sites, immediately downstream of the luciferase reporter gene. The mutant 3′-UTRs were established via introducing seven mismatched mutations into the putative seed regions of HO-1.

The sequences of wild type and mutant HO-1 3′-UTR were as follows:

Wild: TGGAAGGCCTTCTTTCTAGAGAGGGAATT
Mutant: TGGAAGGCCTTCTTAGAAGAGAGGGAATT

All constructs were sequenced to verify that the 3’-UTR was successfully inserted. The OASFs were temporarily transfected with HO-1 3′-UTR luciferase plasmids via Lipofectamine 2000 following the manufacturer’s instructions.

### Statistical analysis

All of the continuous variables are expressed as mean ± standard deviation (SEM). The Mann-Whitney U test was utilized for non-parametric distributions. The *p* value of less than 0.05 was considered statistically significant.

## Results

### TGF-β1 stimulated the expression of HO-1 in human OASFs

**Fig 1:**
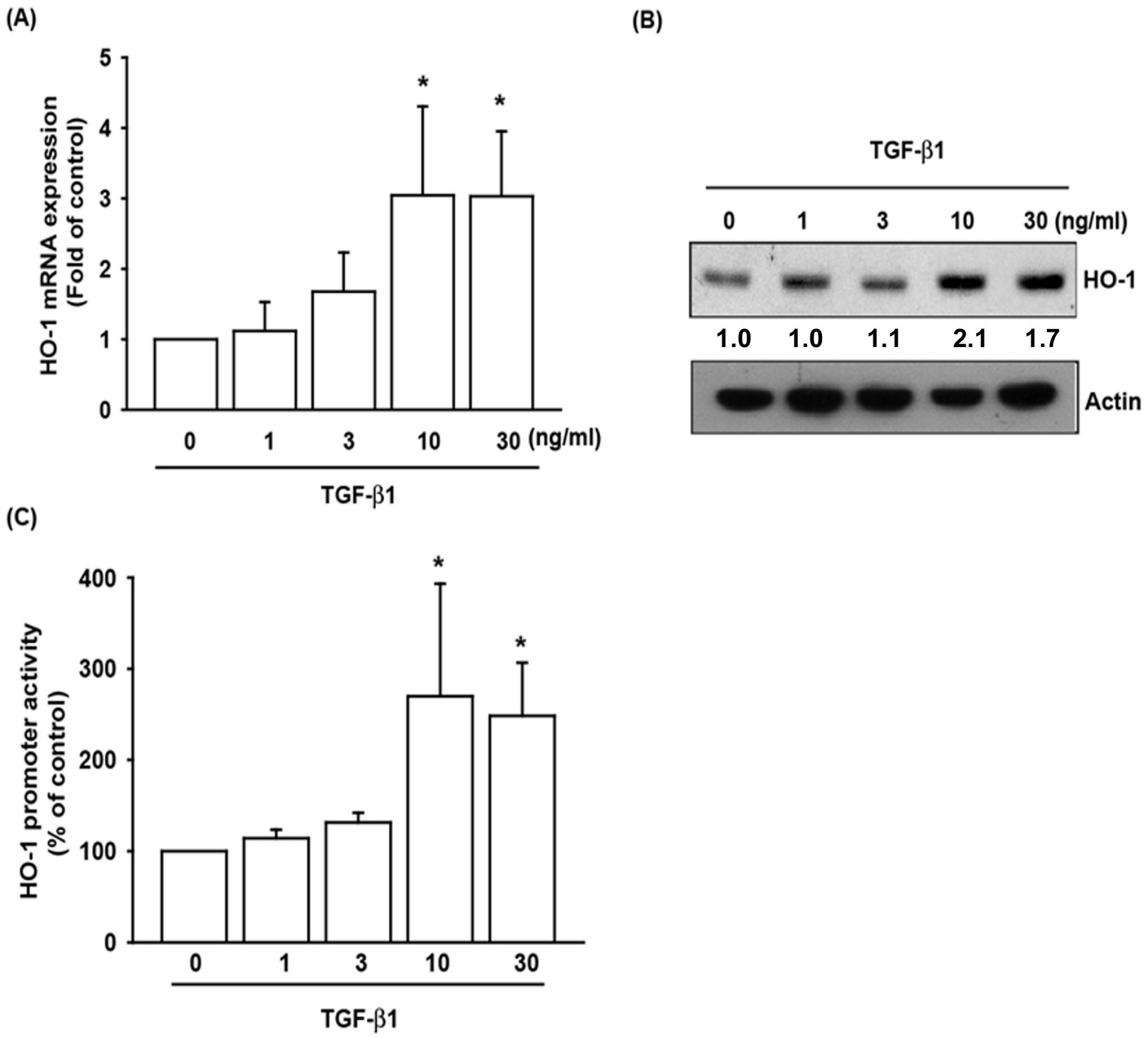
TGF-β1 stimulates HO-1 expression in OASFs in a concentration-dependent manner. (A) Human OASFs were incubated with 0, 1, 3, 10, and 30 ng/ml of TGF-β1 for 24 hours, and HO-1 mRNA expression levels were examined by qPCR (n = 4 per group). (B) OASFs were incubated under various concentrations of TGF-β1 for 24 hours, and HO-1 expression levels were examined by Western blotting (n = 3 per group). (C) OASFs were transfected with the HO-1-luciferase expression vector and incubated with various concentrations of TGF-β1 for 24 hours. The Luciferase intensity reflects HO-1 promoter activity (n = 4 per group). *: p < 0.05 as compared with the control group.

**Fig 2:**
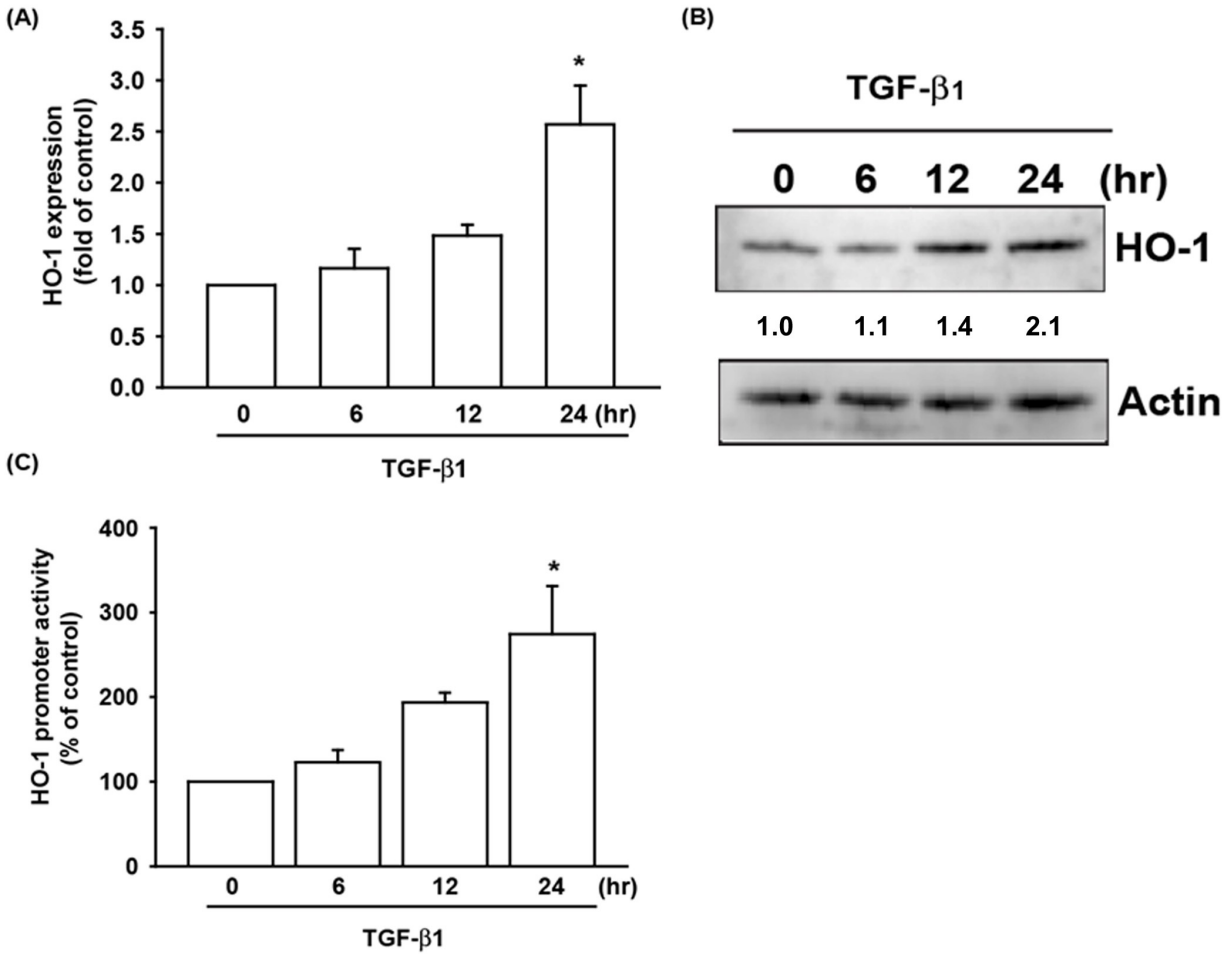
Time-dependent increases in HO-1 expression after TGF-β1 administration. (A) OASFs were incubated with 10 ng/ml of TGF-β1 for 0, 6, 12, and 24 hours. HO-1 mRNA levels were examined by qPCR (n = 4 per group). (B) HO-1 protein synthesis levels were examined by Western blotting (n = 3 per group). (C) OASFs were transfected with the HO-1-luciferase expression vector for 24 hours and then incubated with 10 ng/ml of TGF-β1 for several hours. HO-1 luciferase activity was assayed (n = 4 per group). *: p < 0.05 as compared with the control group.

Both TGF-β1 and HO-1 have been shown to act as anti-inflammatory mediators in previous studies [6, 9]. However, the cross-talk between TGF-β1 and HO-1 in the pathogenesis of OA and their impact on OASFs has been poorly understood to date. We found that TGF-β1 (0 to 30 ng/ml) stimulated the synthesis of HO-1 protein by OASFs in a concentration-dependent manner (Fig 1A and 1B). TGF-β1 also enhanced HO-1 promoter activity in the concentration-dependent manner in the reporter luciferase assay (Fig 1C). When we treated OASFs with TGF-β1 (10 ng/ml) for 0 to 24 hours, we found that TGF-β1 stimulated the synthesis of HO-1 protein in a time-dependent manner under the Western blotting and ELISA assay (Fig 2A and 2B). The relative luciferase activity of OASFs transfected with reporter plasmid showed time-dependent increases after treatment with TGF-β1 (10 ng/ml) from 0 to 24 hours (Fig 2C). These results indicate that TGF-β1 enhances downstream expression of HO-1 in human OASFs, in a concentration- and time-dependent manner.

### TGF-β1 stimulated HO-1 expression via the phosphorylation of PLCγ and PKCα

**Fig 3:**
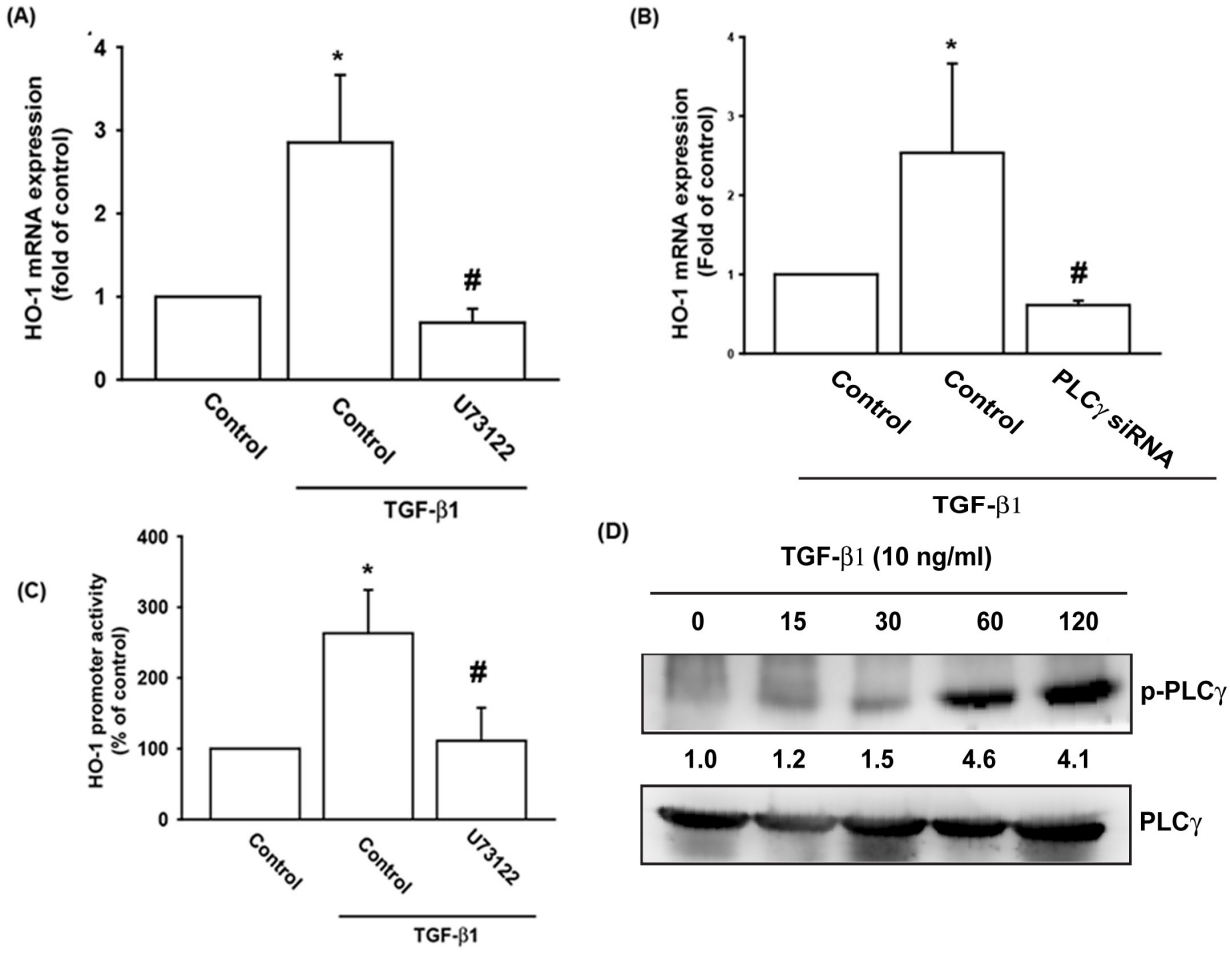
PLCγ activity is involved in TGF-β1-induced HO-1 synthesis. (A&B) OASFs were pretreated with a PLC inhibitor (U73122; 10 μM) for 30 minutes or transfected with PLCγ siRNA for 24 hours, then incubated with TGF-β1 (10 ng/ml) for 24 hours. HO-1 mRNA levels were examined by qPCR (n = 4 per group). (C) OASFs were transfected with the HO-1-luciferase expression vector and then pretreated with U73122 before incubation with TGF-β1 for 24 hours. Luciferase activity was assayed (n = 4 per group). (D) OASFs were incubated with TGF-β1 for the indicated time intervals, and the extent of PLCγ phosphorylation was examined by Western blotting (n = 3 per group). *: p<0.05 as compared with baseline. #: p<0.05 as compared with the TGF-β1-treated group.

The PLCγ/PKCα signaling cascade plays a substantial role in cellular functions that are triggered by various stimuli, including TGF-β1. To investigate the signaling pathway involved in TGF-β1-enhanced HO-1 production, OASFs were pretreated with a PLC inhibitor (U73122) or transfected with PLCγ siRNA. Western blotting analysis and ELISA assay confirmed significant elimination of TGF-β1-enhanced HO-1 protein synthesis in OASFs, as shown in Fig 3A and 3B. Pretreatment of OASFs with U73122 attenuated TGF-β1-enhanced HO-1 promoter activity (Fig 3C). Stimulation of OASFs with TGF-β1 led to an apparent increase in phosphorylation of PLCγ as determined by a Western blotting assay (Fig 3D).

**Fig 4:**
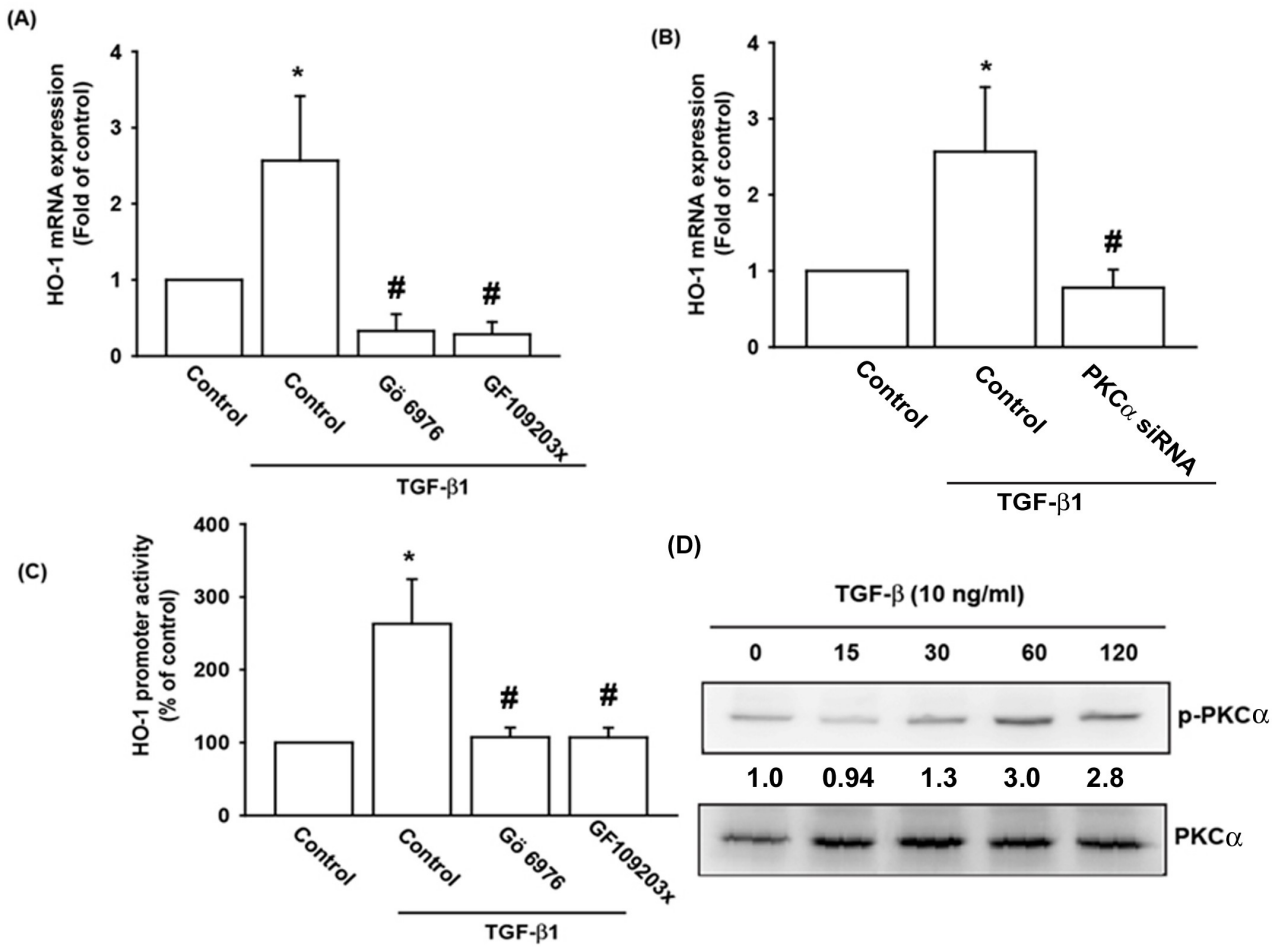
PKCα is involved in TGF-β1-induced HO-1 production. (A&B) OASFs were pretreated with a PKC inhibitor (GF109203x; 10 μM) and a specific PKCα/β inhibitor (Gö6976; 10 μM), or transfected with PKCα siRNA for 24 hours, then incubated with TGF-β1 (10 ng/ml) for 24 hours. HO-1 mRNA levels were examined by qPCR (n = 4 per group). (C) OASFs were transfected with the HO-1-luciferase expression vector then pretreated with Gö6976 and GF109203x before incubation with TGF-β1 for 24 hours. Luciferase activity was assayed (n = 4 per group). (D) OASFs were incubated with TGF-β1 for indicated time intervals, and the extent of PKCα phosphorylation was examined by Western blotting (n = 3 per group). *: p<0.05 as compared with baseline. #: p<0.05 as compared with the TGF-β1-treated group.

PLC hydrolyzes phosphatidylinositol 4, 5-bisphosphate (PIP_2_), producing diacyl glycerol (DAG) and inositol 1, 4, 5-triphosphate (IP_3_). DAG, in concert with calcium ion, activates PKC[18]. In previous study indicated that PKCα is involved in regulating HO-1 expression[19]. Similar methodology was utilized to elucidate the effects of PKCα. OASFs were pretreated with a PKC inhibitor (GF109203x), a specific PKCα/β inhibitor (Gö6976) and PKCα siRNA before TGF-β1 administration. As shown in Fig 4A and 4B, pretreatment with GF109203x and Gö6976, or transfection with PKCα siRNA, significantly eliminated TGF-β1-enhanced HO-1 expression. In accordance with the findings mentioned above, pretreatment of OASFs with GF109203x and Gö6976 curtailed the TGF-β1-enhanced HO-1 promoter activity (Fig 4C). Furthermore, TGF-β1 stimulated PKCα phosphorylation, as shown in Western blotting analysis (Fig 4D). These combined results demonstrate that TGF-β1 enhances HO-1 expression by stimulating the phosphorylation of PLCγ/PKCα.

### TGF-β1 enhanced HO-1 expression by inhibiting miRNA-519b synthesis

**Fig 5:**
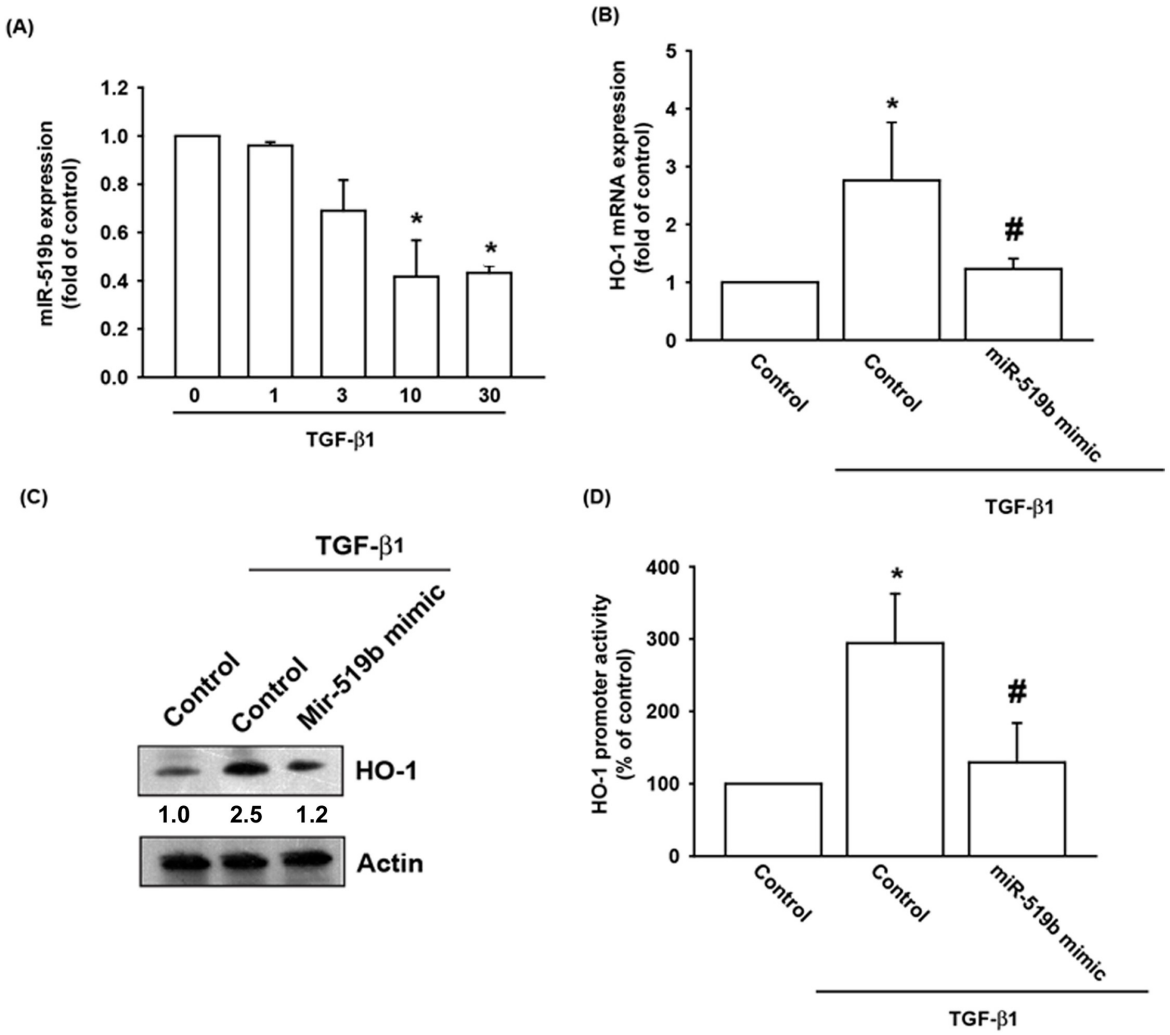
TGF-β1 suppression of miRNA-519b enhances HO-1 production. (A) OASFs were incubated with TGF-β1 for 0, 1, 3, 10, and 30 ng/ml. miRNA-519b expression levels were examined by qPCR assay (n = 4 per group). (B) OASFs were transfected with miR-519b mimic and stimulated with TGF-β1 for 24 hours. HO-1 mRNA expression was examined by qPCR assay (n = 4 per group). (C) OASFs were transfected with miRNA-519b mimic then stimulated with TGF-β1 for 24 hours. HO-1 expression levels were examined by Western blotting (n = 3 per group). (D) OASFs were co-transfected with miR-519b mimic and the HO-1-luciferase expression vector, then pretreated with Gö6976 and GF109203x before incubation with TGF-β1 for 24 hours. Luciferase activity was assayed (n = 4 per group). *: p<0.05 as compared with baseline. #: p<0.05 as compared with the TGF-β1-treated group.

Several miRNAs demonstrate differential expression patterns between osteoarthritic and normal cartilage and are involved in the inflammatory and catabolic processes of OA [20, 21]. However, the precise roles of miRNAs in the pathogenesis of OA remain far from clear. Open-source software (TargetScan, miRDB, and miRWalk) was utilized in this study to identify the miRNAs that could possibly interfere with the transcription of HO-1. The findings showed that the 3’UTR region of the HO-1 promoter harbors potential binding sites for 20 candidate miRNAs, and miRNA-519b is downregulated to the greatest extent after TGF-β1 stimulation (data not shown). To confirm these findings, we directly compared the expression levels of miRNA-519b in OASFs with and without TGF-β1 administration. We found that TGF-β1 (1 to 30 ng/ml) inhibited miRNA-519b expression in a concentration-dependent manner (Fig 5A). To further determine if TGF-β1 stimulates HO-1 expression by inhibiting miRNA-519b synthesis, OASFs were transfected with the miRNA-519b mimic, which subsequently diminished TGF-β1-enhanced HO-1 mRNA and protein synthesis (Fig 5B and 5C). In addition, treatment of OASFs with the miRNA-519b mimic attenuated TGF-β1-induced HO-1 promoter activity (Fig 5D). These findings suggest that TGF-β1 promotes HO-1 expression by suppressing miRNA-519b expression in OASFs.

**Fig 6:**
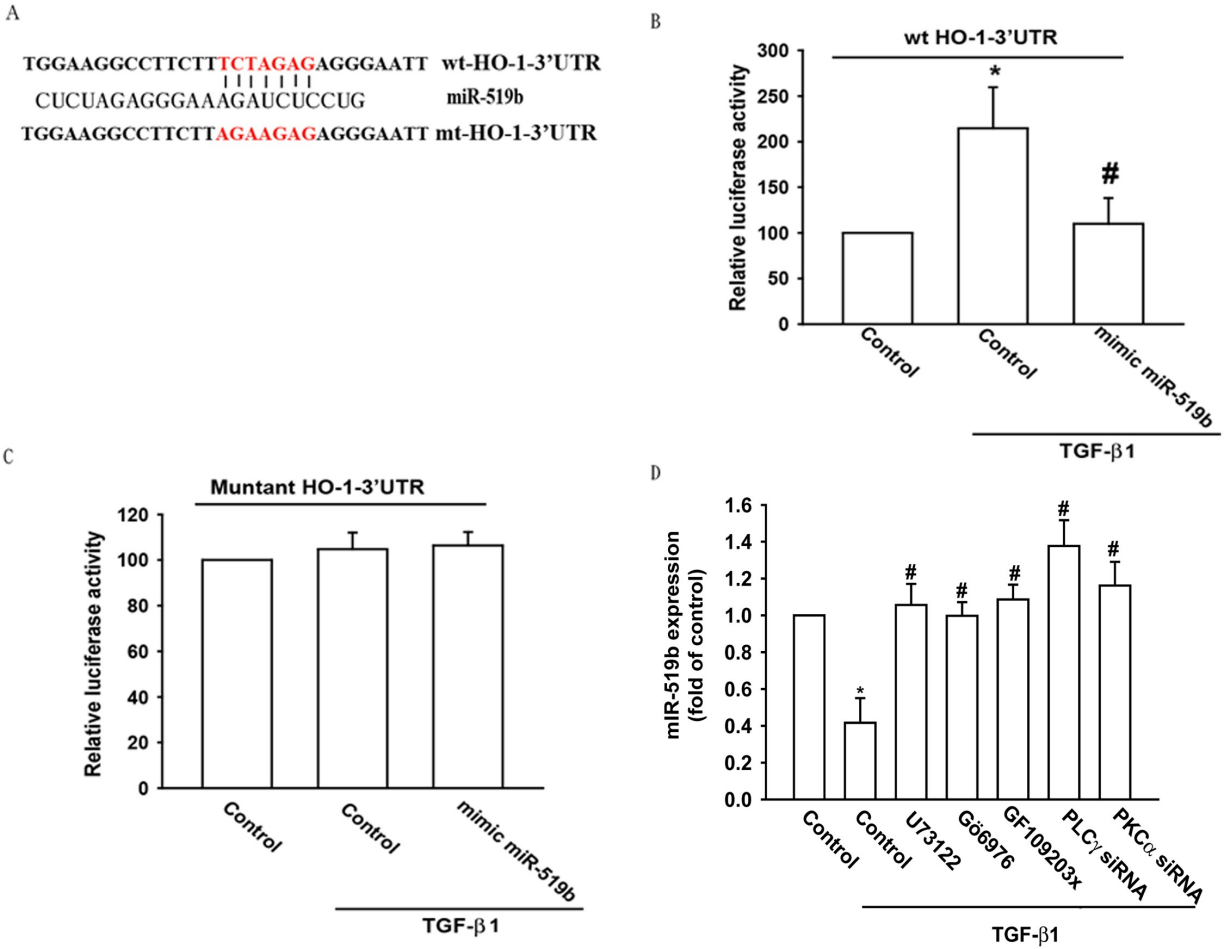
The binding of miRNA-519b to HO-1 3’ UTR mitigates TGF-β1-induced increases in HO-1 expression. (A) Diagram of the miRNA-519b binding site in the wild-type and mutant HO-1 3′UTRs. In order to evaluate miRNA-519b binding, the 3′UTR mutant was used. The “full mutant” lost all apparent binding of miRNA-519b. Cells were co-transfected with miRNA-519b and the wt-HO-1-3′UTR or mt-HO-1-3′UTR plasmids for 24 hours, and the relative luciferase activity was measured. (B) OASFs were transfected with the wt-HO-1-3′UTR plasmid with or without miRNA-519b mimic, then stimulated with TGF-β1. HO-1 promoter activity was expressed as the relative luciferase activity (n = 3 per group). (C) OASFs were transfected with the mt-HO-1-3′UTR plasmid with or without miRNA-519b mimic, then stimulated with TGF-β1. The relative luciferase activity reflected HO-1 promoter activity (n = 3 per group). (D) OASFs were pretreated with U73122, Gö6976 and GF109203x for 30 min or transfected with PLCγ and PKCα siRNA for 24 hours, then incubated with TGF-β1 (10 ng/ml) for 24 hours. MiR-519b expression was examined by qPCR (n = 4 per group). *: p<0.05 as compared with baseline. #: p<0.05 as compared with the TGF-β1-treated group.

We also used the luciferase reporter vector including the wild-type 3′UTR of HO-1 mRNA (wt-HO-1-3′UTR) as well as the vector harboring mismatches in predicted miRNA-519b binding site (mt-HO-1-3′UTR) to determine whether miRNA-519b regulates the 3’UTR of HO-1 mRNA (Fig 6A). Fig 6B shows that miRNA-519b mimic reduced TGF-β1-enhanced luciferase activity in the wt-HO-1-3′UTR plasmid but not in the mt-HO-1-3′UTR plasmid (Fig 6B and 6C). In addition, the PLC inhibitor (U73122) and the PKC inhibitors (GF109203x and Gö6976) or PLCγ and PKCα siRNA reversed TGF-β1-inhibited miRNA-519b expression (Fig 6D). These data suggest that miRNA-519b directly suppresses HO-1 gene transcription via binding to the 3’UTR region of the human HO-1 gene promoter, and that miRNA-519b expression is negatively regulated by PLCγ/PKCα phosphorylation induced by upstream TGF-β1 signaling.

## Discussion

The role of TGF-β1 in OA pathogenesis is multi-factorial. TGF-β1 induces synovial fibrosis via the Smad dependent and Smad independent pathways [22] and has been recognized as having unique, potent anti-inflammatory properties in arthritic diseases. TGF-β1 is synthesized in the synovial fluid of patients with rheumatoid arthritis (RA), and is correlated with disease remission. Overexpression of the TGF-β1 gene mitigates the severity of arthritis in animal models [23–25]. Despite the major role played by TGF-β1 in the pathogenesis of OA and the anti-inflammatory properties, the impact of TGF-β1 on osteoarthritic synovial tissues has not yet been defined. Our study findings show that TGF-β1 induces the expression of several anti-inflammatory mediators of synovial cells; levels of HO-1 expression are the most apparent. We demonstrate that TGF-β1 enhanced the expression of HO-1 via the stimulation of PLCγ/PKCα phosphorylation and suppression of the downstream expression of miRNA-519b, an miRNA that disrupts the transcription of the HO-1 gene. These results may add to the literature on OA pathogenesis.

The function of enhanced expression of HO-1 by OASFs after long-time induction of stimulatory molecules (ex: TGF-β1) is unknown. However, the constitutively enhanced HO-1 expression in the Bach 1 gene knockout mice could lead to reduced apoptosis of chondrocytes and reduced severity of OA, increased expression of autophagy marker, and increased activity of anti-oxidant enzymes[26]. The beneficial results from the animal model with constitutively enhanced HO-1 expression may be extrapolated to infer the implication of the induction of HO-1 expression by OASFs after a long-time exposure of TGF-β1.

The miRNAs are small, non-coding RNA segments modulating the post-transcriptional regulation of gene expression in multi-cellular organisms by suppressing the translation of or degrading the target mRNAs[27]. The miRNA-146, miRNA-155 and miRNA-203 have been reported to be involved in OA pathogenesis [28–30]. We used open-source software (TargetScan, miRDB, and miRWalk) to compare the miRNA expression profiles of OASFs before and after TGF-β1 administration and found that miRNA-519b was apparently suppressed by TGF-β1. This decrease in miRNA-519b expression was confirmed by qPCR assay. We have shown that transfection of OASFs with miRNA-519b mimic attenuates TGF-β1-stimulated HO-1 expression, while the luciferase assay showed that mutant HO-1 promoter activity was enhanced by TGF-β1 administration. These findings underscore the importance of miRNA-519b in the process of TGF-β1 stimulation of HO-1 synthesis.

The role of the PLCγ/PKCα pathway in the pathogenesis of arthritic diseases has been discussed previously [31]. The proliferation of synoviocytes from patients with RA has been reported to be suppressed by PLC and PKC inhibitors [31]. In our previous research, we found that the PLC/PKCα pathway was involved in thrombin-induced interleukin-6 synthesis in rheumatoid synovial cells [32]. Previous studies have suggested that TGF-β1 regulates the inward rectifier potassium channel via the PLC/PKCα pathway in reactive astrocytes and adult rat brain [33]. Linkage of the PLC/PKCα pathway and downstream HO-1 expression has been demonstrated in mouse brain endothelial cells [34]. In this study, we show that TGF-β1 stimulates HO-1 synthesis via PLCγ/PKCα phosphorylation. These results augment those from previous studies and highlight the importance of the PLCγ/PKCα pathway in the pathogenesis of arthritic diseases.

Our study shows that the binding of TGF-β1 in OASFs triggers the phosphorylation of PLCγ and PKCα, contributing to the suppression of miRNA-519b synthesis. The subsequent decrease in miRNA-519b expression enhances HO-1 promoter activity and HO-1 protein synthesis. These results may elucidate understanding about the role of OASFs in the pathogenesis of OA and lead to novel and effective therapy for patients with OA.
